# Facing the Shadow Pandemic: Correlation and Trend Analyses of Violence Reports from Women to the Italian National Anti-Violence Number during 2021

**DOI:** 10.3390/healthcare11162272

**Published:** 2023-08-11

**Authors:** Antonio Del Casale, Jessica Pileri, Giorgio Veneziani, Antonio Napolitano, Martina Nicole Modesti, Barbara Adriani, Benedetta Barchielli, Giovanna Parmigiani, Francesco Grassi, Christian Napoli, Stefano Ferracuti, Carlo Lai

**Affiliations:** 1 Department of Dynamic and Clinical Psychology and Health Studies, Faculty of Medicine and Psychology, Sapienza University of Rome, 00189 Rome, Italy; jessica.pileri@uniroma1.it (J.P.);; 2 Unit of Psychiatry, Sant’Andrea University Hospital, 00189 Rome, Italy; 3 Scientific Institute for Research, Hospitalization and Healthcare “Bambino Gesù”, 00146 Rome, Italy; 4 Department of Psychology, Faculty of Medicine and Psychology, Sapienza University of Rome, 00185 Rome, Italy; 5 Department of Neuroscience, Mental Health and Sensory Organs (NESMOS), Faculty of Medicine and Psychology, Sapienza University of Rome, 00189 Rome, Italy; 6 Department of Medical-Surgical Sciences and Translational Medicine, Faculty of Medicine and Psychology, Sapienza University, 00189 Rome, Italy; 7 Unit of Risk Management, Sant’Andrea University Hospital, 00189 Rome, Italy; 8 Department of Human Neuroscience, Faculty of Medicine and Dentistry, Sapienza University of Rome, 00185 Rome, Italy

**Keywords:** COVID-19 pandemic, interpersonal violence, quarantine, battered women, mental health

## Abstract

Objectives: To help to tackle gender-based violence against women in the aftermath of COVID-19 or other potential crisis situations, as recommended by the European Institute for Gender Equality, the present study aims to investigate the relationship between calls to the National Anti-violence Number (NAN) and the socio-sanitary pandemic factors during 2021, a period in which the scenario changed due to containment measures that gradually allowed women to emerge from the condition of isolation. In addition, the present work aims to identify significant changes in the number of calls to NAN during the progression of the pandemic in 2021. Methods: Using Italian Civil Protection Department data on the socio-sanitary pandemic factors (defined by daily number of cases, swabs, deaths, hospitalizations, dismissions, intensive care unit hospitalizations, people isolated at home, and quarantine after 15 and 30 days) and NAN data. Correlation analyses, a joinpoint regression analysis, and Granger causality tests were performed. Results: The number of calls correlated with the daily number of cases, swabs, deaths, hospitalizations, dismissions, intensive care unit hospitalizations, and quarantine after 15 and 30 days. The identified joinpoints showed significant changes in the number of daily anti-violence calls during the year. Furthermore, we found evidence of a significant causality between daily hospitalizations, daily home quarantined, daily dismissed patients, and calls to NAN. Conclusions: The results underline the influence of containment measures on the increased access to NAN, suggesting the need for a greater implementation of social and psychological support services in other potential crisis situations.

## 1. Introduction

Gender-based violence (GBV) against women is a phenomenon that includes physical, sexual, and psychological violence directed against women, including threats of such acts, coercion, and deprivation of liberty [1]. GBV against women is a global health and social problem with harmful health, social, and economic consequences [2]. Undoubtedly, GBV against women can be identified as a true social problem. A social problem exists when an influential group is aware of a social condition that challenges its values and can be addressed through collective action [3]. Additionally, Rubington and Weinberg [4] described it as an alleged situation that contradicts the values of a significant number of individuals who recognize the need for action to rectify the situation. Research in this area is crucial as it holds significance within the production of knowledge in a collective dimension to implement protective measures on a broader scale. 

Internationally, the COVID-19 pandemic control measures have exacerbated women’s experiences of all forms of violence [5]. According to UNFPA [6], pandemics often lead to social infrastructure breakdowns, thus compounding the existing weaknesses and conflicts. Indeed, across Europe, the measures taken by states to address the pandemic have increased the vulnerability and risk of violence for women [7]. Social and economic stress has amplified pre-existing social norms based on gender inequality [8]. In this context, it has been reported that stress derived from precarious economic situations could increase the probability of intimate partner violence [9] and reduce protective social networks [10]. Furthermore, previous studies have traced, among the causes of the increase in GBV against women, the greater conditions of vulnerability attributable to quarantine, isolation, social distancing, and movement limitations, derived from the measures implemented to counter the COVID-19 pandemic [10,11,12]. Indeed, recognizing the safety risks associated with lockdowns, in April 2020, the United Nations labeled violence against women a “shadow pandemic” [13]. This becomes even more noticeable, considering that the prevalence of domestic violence seems to be underestimated; for example, in Western countries, 25% of women experience domestic violence, but only 2.5–15% of women report it [14,15].

In 2020, Italy was one of the first European nations to experience widespread virus transmission and adopt drastic measures to contain its spread. These measures had a significant impact on the economy and daily life of Italian citizens. During this period, the number of COVID-19 cases and deaths was very high. The restrictive measures adopted in 2020 focused on implementing generalized measures to control the pandemic at the national level. These measures reached their maximum level of strictness between 9 March and 4 May, during which Italy experienced a period of lockdown. This period saw an increase in episodes of domestic violence compared to 2019 [16]. Over 15,000 women started a personalized path out of violence in 2020 [17]. The data on the worsening of gender-based violence against women are consistent with previous studies, showing that violence increases when faced with a pandemic [10,18]. Interestingly, in Italy, this increase had already been recorded in the case of the plague [19], and, in particular, an increase in gender-based violence has been demonstrated during pandemics and quarantines [20]. In accordance with the European Institute for Gender Equality (2021), it is urgent to understand how to strengthen existing measures and implement new measures to protect and support female victims of intimate partner violence in the aftermath of COVID-19, as well as in other potential crisis situations. Therefore, it is necessary to focus on 2021, when Italy implemented different measures compared to 2020.

In this regard, as for 2021, the initial months maintained some restrictive measures from 2020, with the DPCM (Decree of the President of the Council of Ministers) of the 3 November 2020, which entered into force on the 6 November 2020. The DPCM is a decree with the force of law adopted by the Government in extraordinary cases of necessity and urgency. Italian regions were labeled with different colors (yellow = level of moderate risk; orange = high-risk; red = high criticality) based on the severity of the pandemic in the area. On 14 January 2021, a “white zone” was also established for lower-risk regions. Passing from one level of risk/color to a higher risk one brought more restrictive limitations. Following Legislative Decree No. 56 of the 22 April 2021, a period of recovery gradually began, which included, for example, the gradual development of face-to-face teaching, the possibility of eating in restaurants, albeit outdoors and with capacity limits, and the resumption of various activities in regions labeled as least affected in a given period. Furthermore, the decree-law approved on the 18 May 2021 allowed a series of measures to reduce the curfew, which was definitively eliminated on the 21 June 2021. 

The comparison between the pandemic situation in Italy in 2021 and 2020 highlights a shift from generalized nationwide lockdown measures to a more targeted and flexible approach in 2021. These measures seemed to have allowed women to gradually emerge from a situation of isolation, a condition that, together with others (such as substance use, low income, inability to escape, and reduced services), could exacerbate family violence [16,21]. For women who suffered violence, in Italy, in 2006, the Ministry for Equal Opportunities established the “National Anti-violence Number” (NAN) [22]. Thanks to this number, women who suffer violence can receive support at various levels. As for 2020, previous research has already investigated the phenomenon of domestic violence reports from women during the 2020 pandemic in Italy [23], finding that the number of daily calls from women to NAN was positively correlated with the trend of the pandemic (COVID-19 daily deaths, daily hospitalizations, daily home quarantined, and daily home quarantined postponed by 30 days). However, there is no analysis of violence episodes in 2021, the year in which the scenario changed.

### Gender-Based Violence before and after COVID-19

GBV against women is a pervasive issue that encompasses physical, sexual, and psychological violence, transcending societal boundaries and affecting individuals across different social classes and cultural groups. It is a global problem that has a profound impact on women’s lives, with statistics revealing that approximately one in three women worldwide experience GBV at some point in their lives, even prior to the onset of the COVID-19 pandemic [24]. The prevalence of GBV is alarming, with reports indicating that 35% of women globally have experienced either physical and/or sexual intimate partner violence or non-partner sexual violence [24]. Furthermore, intimate partners are responsible for 38% of female homicides, highlighting the severity of the issue [24]. The situation in Italy regarding GVB against women is aligned with international evidence. According to data from ISTAT [25], approximately 31% of women between the ages of 16 and 70 have experienced physical or sexual violence at least once in their lifetime. It is essential to recognize that GBV knows no age boundaries, affecting women across various stages of their lives [8]. The COVID-19 pandemic and the accompanying lockdown measures have exacerbated pre-existing social norms and gender inequality, further intensifying the issue of GBV against women. During the pandemic, when a substantial portion of the global population was under lockdown, an estimated 243 million women and girls between the ages of 15 and 49 experienced sexual and/or physical violence perpetrated by an intimate partner [13]. The increased pressure on essential services addressing GBV during this time highlights the urgent need for comprehensive support systems.

This becomes even more relevant when considering that the consequences of GBV extend beyond the immediate acts of violence, impacting the physical, mental, and social well-being of victims and resulting in psychological consequences. In addition, these consequences encompass a range of physical, psychological, neurological, and cognitive problems [26,27]. Research has shown that over 50% of individuals who experience GBV develop Post-Traumatic Stress Disorder [28]. GBV can result in various physical (physical symptoms, permanent disabilities, somatic disorders, and gynecological issues), mental (anxiety, depression, phobias, substance abuse, and eating disorders) and fatal outcomes, including homicide and suicide [29]. Recovering from trauma during the COVID-19 pan-demic and the associated lockdowns presents significant challenges. It should also be noted that weaknesses in social and health systems have been amplified, hampering the support and resources needed for survivors [8]. Safety measures implemented during the pandemic have unwittingly exposed GBV survivors to additional risks. Indeed, the intermittent periods of quarantine and lockdown disrupted therapeutic processes and further isolated survivors from their support networks, aggravating their situation [8,28,30]. For these reasons, it appears even more relevant to study the relationships between pandemic aspects and reports to NAN.

Based on these premises, the study has two aims. The first aim is to investigate the associations between the calls received by NAN from women and socio-sanitary pandemic factors. These factors were defined by daily cases (DC), daily swabs (DS), daily deaths (DD), daily hospitalizations (DH), daily intensive care unit hospitalizations (D-ICU-H), daily dismissed patients (DDP), the daily number of people quarantined at home (DHQ), and the daily quarantines postponed (DQP) by 15 and 30 days. It was hypothesized a positive association between these variables and the number of calls to NAN because these variables could have exacerbated the perception of the severity of the pandemic, heightened the fear of a new lockdown, and prompted women to contact NAN.

The second aim is to identify significant changes in the number of calls to NAN during the progression of the pandemic in 2021. A joinpoint regression analysis was conducted to evaluate if there had been a significant change in the number of daily anti-violence calls during 2021.

## 2. Methods

Data on COVID-19 daily cases (DC), daily swabs (DS), COVID-19 daily deaths (DD), COVID-19 daily hospitalizations (DH), COVID-19 daily intensive care unit hospitalizations (D-ICU-H), daily dismissed patients (DDP), the daily number of people isolated at home (referred to as “daily home quarantined”, DHQ), and the daily number of quarantines postponed by 15 and 30 days (DQP) from 1 January 2021 to 31 December 2021 were obtained from the Italian Civil Protection Department of the Presidency of the Council of Ministers [31]. Data on daily calls from women reporting to NAN in 2021 were sourced from the Italian National Institute of Statistics [32]. Data were collected from each Italian Region and supervised by the Istituto Superiore di Sanità (ISS) and the Italian Ministry of Health and Civil Protection Department. The quality of the data, dataset elaboration, and publication procedures were regularly verified on a daily basis [31].

### Statistical Analysis

IBM SPSS Statistics V25.0 (Armonk, NY, USA: IBM Corp) software was used for the descriptive analysis, and correlation tests were conducted among the variables of interest (DC, DS, DD, DH, D-ICU-H, DHQ, DQP, and daily calls from women to NAN). Additionally, trend analyses were performed using Joinpoint Trend Analysis software, Version 4.9.1 (Statistical Methodology and Applications Branch, Surveillance Research Program, National Cancer Institute), and inferential analysis was conducted [33].

For correlations, Kendall’s Tau B statistic was used due to the nonlinearity of associations. Kendall’s Tau-b is suitable for examining non-linear relations [34]. To assess the significance of multiple correlations, Bonferroni corrections were performed. The critical alpha value (0.05) was adjusted for the number of variables included in the multiple correlation. The adjusted alpha (*p* = 0.05/8) value was found to be 0.006.

We used the Granger_Cause_1 [35] function in MATLAB, setting a maximum number of lags of 4. Then, this function chooses the optimal lag length for X and Y based on the Bayesian Information Criterion (BIC) [36]. The Granger causality test adds a causal term to the linear prediction model and compares that to a similar linear model without that term. This comparison of the Residual Sum of Squares was conducted by an F-test, which takes a value near 1 if there is no causality and deviates from 1 if there is a difference in RSS. This value is then translated to a *p*-value using the F-distribution. The Granger causality Test [37] involves using F-tests to test whether lagged information on a variable Y provides any statistically significant information about a variable X in the presence of a lagged X. If not, then “Y does not Granger-cause X”. We considered first the X variable as the daily violence calls from our database and checked if all the variables we had in our database comply with Granger Cause X. We considered a *p*-value that was lower than or equal to 0.1 to be a strong hint towards the alternative hypothesis (causality in this case). To increase the robustness of our results, we used another function available in MATLAB, gctest [38]. The function performs Granger causality tests on all response variables composing the VAR(p) Mdl model created by the varm function. This model stores the estimated parameter values resulting from fitting the VAR(p) Mdl model to all variables of the observed multivariate response series matrix Y using maximum likelihood.

Joinpoints in the context of trend analysis refer to the specific points or periods where a trend is most likely to change and represent significant shifts in the underlying pattern of the data [39]. Joinpoint regression modeling is a statistical technique that allows for the identification and estimation of these joinpoints. Temporal trends in the rate of violence-reporting calls were analyzed using log-linear joinpoint segmented regression models, which identify points corresponding to statistically significant changes over time in the linear slope of the occurring trend [33,40]. The joinpoint regression was estimated at the national level. The daily rates of anti-violence calls were utilized as the dependent variable, assuming homoscedasticity and linearity. To assess significant trends based on the daily percent change (DPC), a log transformation was applied. An uncorrelated error model was employed for the analysis. The range of joinpoint numbers was set from 0 to 5, and a permutation test was performed with an overall significance level set at *p* < 0.05.

## 3. Results

**Figure 1:**
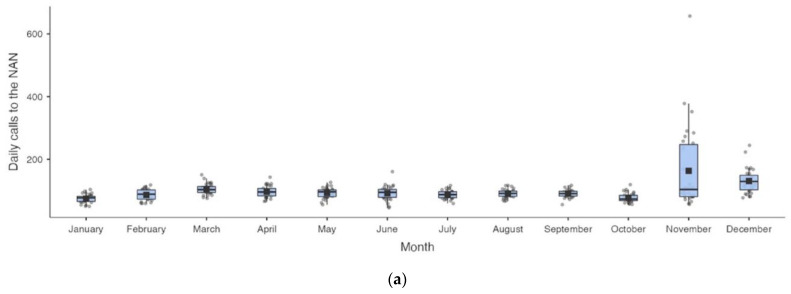

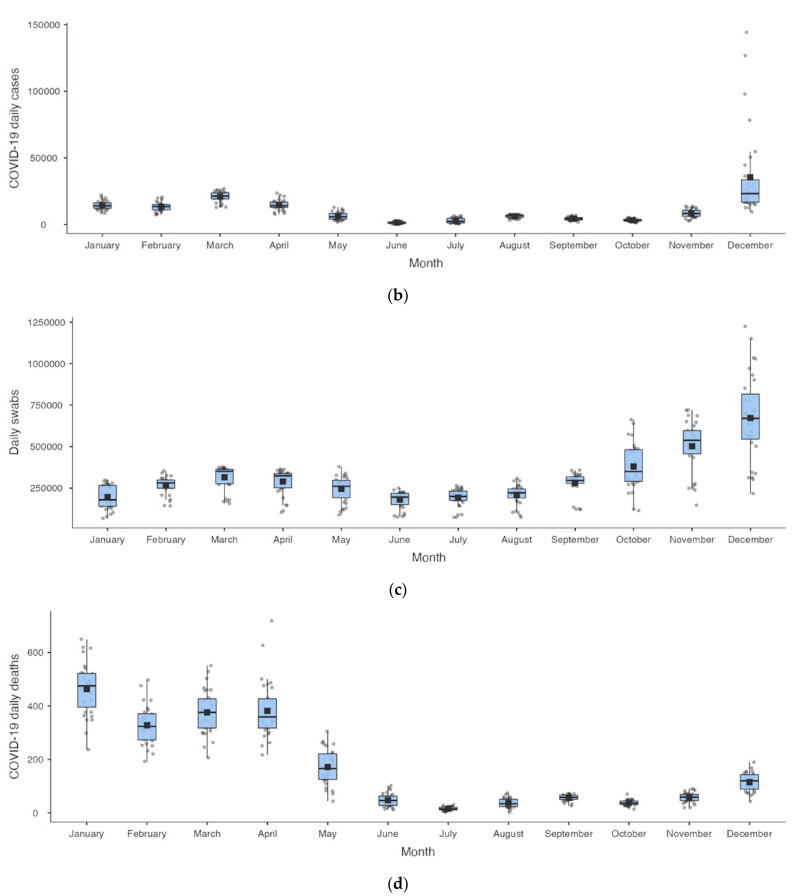

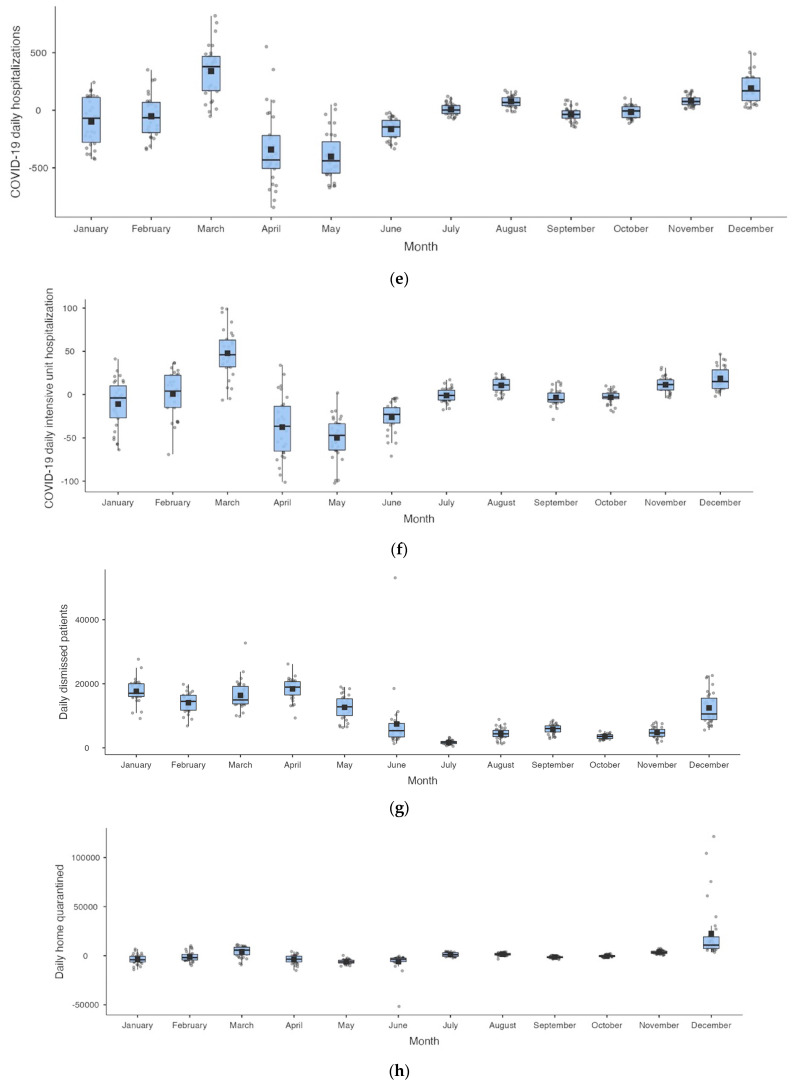

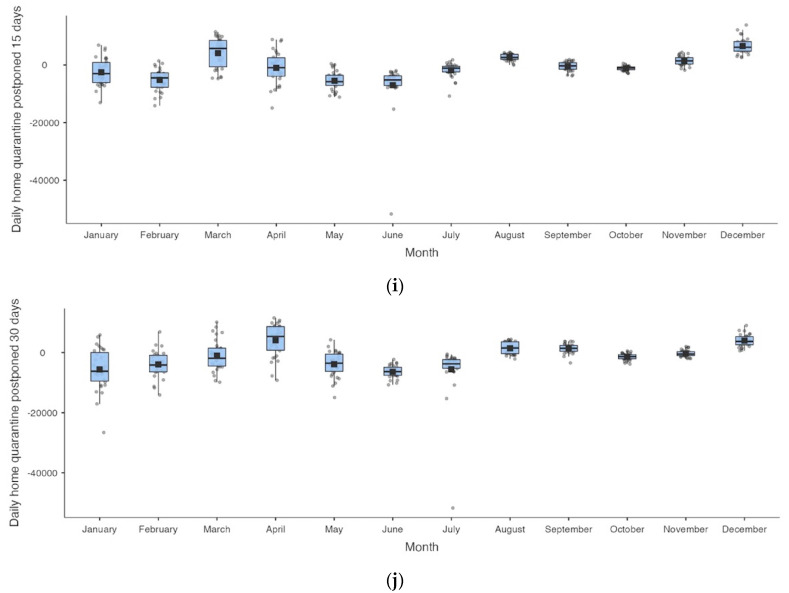
Box plots of the considered variables divided by months. (**a**) Daily calls to the national anti-violence number; (**b**) daily cases (DC); (**c**) daily swabs (DS); (**d**) daily deaths (DD); (**e**) daily hospitalizations (DH); (**f**) daily intensive care unit hospitalizations (D-ICU-H); (**g**) daily dismissed patients (DDP); (**h**) the daily number of people quarantined at home (DHQ); (**i**) the daily quarantines postponed (DQP) by 15; and (**j**) the daily quarantines postponed (DQP) by 30 days.

The main aspects of the study variables, including daily calls to NAN and the considered epidemiological variables, are shown in Figure 1 and Table S1 for each month of 2021 analyzed in the study.

The significance maintained after Bonferroni correction showed that the number of daily calls to NAN positively correlated with the DC (tau-b = 0.258; *p* = 0.001), the DH (tau-b = 0.159; *p* = <0.001), the D-ICU-H (tau-b = 0.105; *p* = 0.003) and DDP (tau-b = 0.111; *p* = 0.002), but not with DS (tau-b = 0.089; *p* = 0.012), the DD (tau-b = 0.089; *p* = 0.012), and the DHQ (tau-b = 0.036; *p* = 0.312), and the number of quarantine cases after 15 days (tau-b = 0.092; *p* = 0.009). Nevertheless, the number of quarantine cases after 30 days positively correlated with the number of daily calls from women’s reports to NAN (tau-b = 0.148; *p* = <0.001) (Table 1).

**Figure 2:**
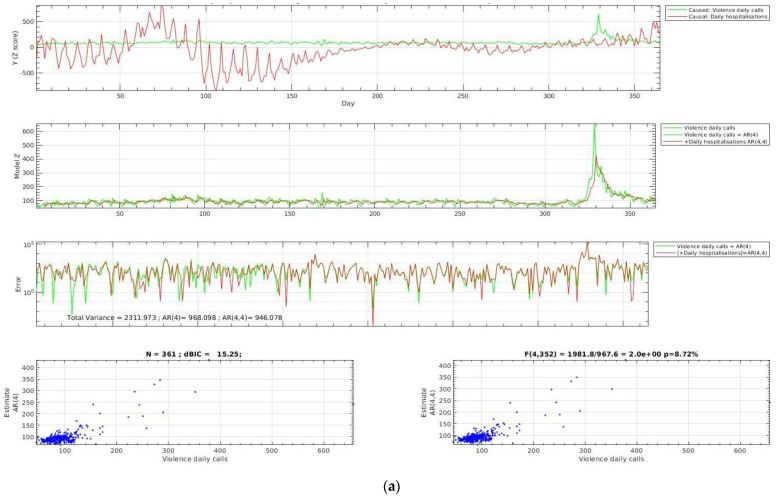

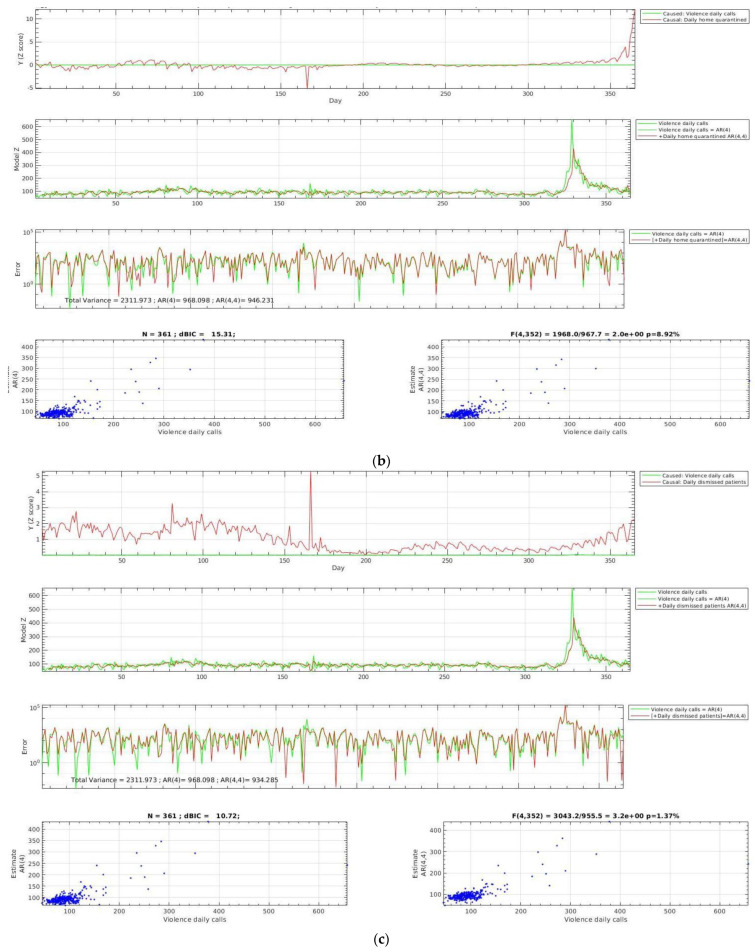
(**a**) Granger causality test: variables Granger causing violence daily calls (daily hospitalizations). (**b**) Granger causality test: variables Granger causing violence daily calls (daily home quarantined). (**c**) Granger causality test: variables Granger causing violence daily calls (daily dismissed patients).

The Granger causality test showed that three variables (daily hospitalizations, home quarantined, and dismissed patients) Granger-caused the variable violence daily calls, and violence daily calls Granger-caused eight variables, i.e., SARS-CoV-2 daily cases, deaths, swabs, hospitalizations, intensive unit hospitalizations, dismissed patients, and quarantined postponed variables (Table 2; Figure 2a–c).

**Figure 3:**
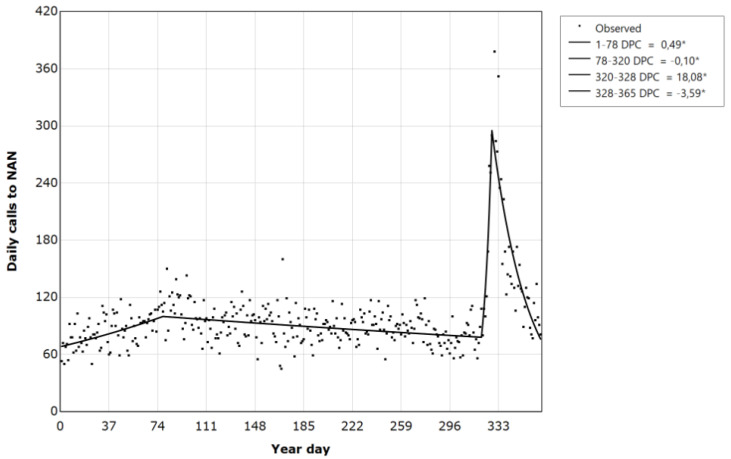
Daily number of calls to the national anti-violence number from 1 January 2021 to 31 December 2021. * Indicates that the daily percent change (DPC) significantly differs from zero at the alpha = 0.05 level.

Joinpoint regression analyses for the year 2021 showed that the cases of calls to NAN showed a stable trend, with an average daily percent change of 0 (t = 0.3; *p* = 0.791). A model with three joinpoints revealed a period from 1 January to 19 March with a daily percent change (DPC) increase of 0.5 calls (DPC = 0.5; t = 4.9; *p* < 0.001), followed by a significant decrease from 19 March to 16 November (DPC = −0.1; t = −5.6; *p* < 0.001), a significant increase from 16 to 24 November (DPC = 18.08; t = 3.5; *p* < 0.001), and a significant decrease until 31 December (DPC = −3.59; t = −11.9; *p* < 0.001) (Table 3 and Figure 3).

## 4. Discussion

The present study aimed to investigate the associations between calls from women reporting to the National Anti-violence Number and socio-sanitary pandemic factors (defined by daily cases (DC), daily swabs (DS), daily deaths (DD), daily hospitalizations (DH), daily intensive care unit hospitalizations (D-ICU-H), daily dismissed patients (DDP), the daily number of people quarantined at home (DHQ), and the daily quarantines postponed (DQP by 15 and 30 days) and to identify significant changes in the number of calls during the evolution of the pandemic in 2021. The results show a positive correlation between the daily number of calls to the NAN and DC, DH, D-ICU-Hm and DQP by 30 days, but not with DS, DD, DHQ, and DQP by 15 days. Furthermore, Granger causality links between daily hospitalizations, daily home quarantined, daily dismissed patients, and calls to NAN were found.

Contrary to what was found in 2020 [23], DD was not significantly related to the daily number of calls in 2021. It could be hypothesized that, during 2020, the number of deaths indicated the severity of the pandemic, as it was at the center of media communication, explaining the established containment measures, such as the lockdown period, but in 2021 the number of deaths was not a criterion for the transition of the Italian Regions’ status from one color to another, and therefore not directly influential on the risk of being in a region with more restrictive measures, which entailed a greater compulsory stay at home.

Survivors of domestic violence have traced partner abuse and control techniques to the restrictive measures derived from the COVID-19 pandemic [41]. The condition of isolation indeed trapped victims with their aggressors who could implement coercive and abusive tactics [42,43,44]. Therefore, factors associated with fear of stricter containment measures may have prompted women to call NAN. This reasoning probably also explains the significant correlation between the daily number of calls to NAN and DH and D-ICU-H. In particular, the link between hospitalization and calls to NAN is also confirmed by the significance of the Granger causality test, suggesting that DH could increase the number of calls to NAN. The hospitalization rate was the only criterion for moving from one area of risk severity to another throughout the year, and for this reason, it may have increased the fear of a new confinement at home. Similarly, the number of cases has been a criterion for at least 6 months [45] and, probably for this reason, DC numbers significantly correlate with calls to NAN.

Another significant correlation was found in relation to DDP and NAN calls. This link also found support in the Granger causality test. It should be kept in mind that the number of patient discharges is also to be considered a social health variable that is part of the disease and hospitalization process with consequent hospital discharge, and therefore can be configured as an index of pandemic severity. In addition, some women may have called out of fear of repeat episodes due to the perpetrators being discharged from the hospital, or may have been discharged from the hospital themselves and therefore called to protect themselves once they return home.

In our scientific investigation, we established a causal link between DHQ and the frequency of calls to NAN. Moreover, our analysis revealed a positive correlation between the number of quarantine cases after 15 and 30 days and the daily volume of calls made to NAN. Female victims of violence may have had vicarious or direct contact with home-extended quarantines, making a new situation of isolation and conditioning within the home possible or effective, especially for longer quarantines. How people respond to risks is influenced by how vividly they can recognize consequences [46], so the resulting perception of danger could explain the association with the number of calls to NAN.

We further substantiated our findings with evidence of inverse causality, indicating that various pandemic variables tend to increase after a rise in daily calls to NAN. This intriguing observation suggests a potential indication of emerging aggressive behaviors before the diagnosis of COVID-19 or concerns surrounding the discharge process and the readjustment to daily life post-quarantine. The context of the SARS-CoV-2 pandemic highlights that exposure to COVID-19 may elicit negative emotions, such as anxiety, a recognized risk factor for aggressive behaviors [47,48]. COVID-19 itself can be associated with delirium [49,50], agitation, and symptoms of depression, anxiety, and insomnia, [51], together with obsessive–compulsive behaviors [52], which could exacerbate aggressive behavior; aggressiveness and violence themselves have been identified as possible manifestations of SARS-CoV-2-unmasked psychiatric conditions, such as, potentially, manic episodes [53,54] 

The joinpoint regression results show that joinpoints can be related to important moments. The first joinpoint corresponds to the 78th day, or 19 March, in correspondence with a period of uncertainty but after a progressive improvement in the situation, which led, about a month later, to the first provisions to loosen the virus containment measures. joinpoint 2, which corresponded to the 328th day analyzed, or 16 November, was a period of the pandemic in which, following a re-increase in infections, there was talk of implementing before Christmas the “Saving Christmas plan”, as the media defined it. Traditionally, when forced proximity increases, Christmas and other holidays are associated with increased domestic violence [55,56]. It can be hypothesized that the fear of spending both the holidays and the previous period with a strengthening of the confinement measures may have activated emotions attributable to fear, which laid the foundations for the increase in calls to NAN.

The call increase peaked in joinpoint 3, or the 328th day (24 November). On 25 November, in Italy, awareness was raised on the theme of GBV against women with the establishment of the “International Day for the Elimination of Violence against Women”. Awareness campaigns often begin before this date by providing information on dedicated appointments and events and sponsoring NAN. The increase in calls around that date could be derived from the greater media visibility of the anti-violence services and the telephone number in question. ISTAT [22] also reported that the days around 25 November recorded increased calls to NAN. However, this significant increase in calls was not recorded in 2020. Further studies are needed to address if the different types of restrictive measures adopted in 2021 may have favored an increase in requests for help, probably due to the lesser surveillance of the aggressors, who had greater freedom [57] and less probability of limiting victims’ access to phones or the Internet [58].

Considering the results of the present study, it seems extremely relevant to implement psychological support services and consider these phenomena in view of possible future containment measures caused by pandemics or other emergencies. More specific and targeted containment measures allowed women to ask for more help. In addition to these modifiable factors at the legislative and institutional levels, further suggestions can be made. According to Rieger et al. [44], addressing GBV during pandemics requires developing and implementing brief screenings or confidential reporting mechanisms for abuse. These measures can help in the timely identification and support of GBV survivors. Additionally, it is crucial to establish resources and emergency daycare facilities, in addition to emergency housing, to provide necessary support to survivors. These initiatives should be implemented alongside the development of brief screenings, allowing for the confidential reporting of abuse. By adopting these strategies, it is possible to enhance the response to GBV and ensure the safety and well-being of survivors during the pandemic or other emergencies.

### Limitations

The current study did not include an examination of socio-demographic factors and the reasons behind calls made to NAN because the authors did not have access to this data. Nevertheless, the study also focused on trend analyses, which did not require the aforementioned assessments. Among the limitations, it should also be noted that calls to NAN may not fully represent the number of women experiencing violence. In fact, during lockdown, women faced difficulties in accessing support while being confined with their abusers, potentially resulting in an underreporting of violence cases. The characteristics of women able to seek help may differ from those who could not, highlighting the complexity of the situation. 

## 5. Conclusions

This study showed that the number of calls to NAN correlated with the daily number of cases, swabs, deaths, hospitalizations, dismissions, intensive care unit hospitalizations, and quarantine after 15 and 30 days. There was a significant causality between daily hospitalizations, daily home quarantined, daily dismissed patients, and the trend of calls to NAN, and it was found to be associated with the implementation of various pandemic containment measures. Furthermore, these calls followed a peculiar trend in 2021, which is probably attributable to risk perception concerning the tightening of containment measures. This study identified three significant points in the analysis timeline. The first point, corresponding to 19 March, marked a period of uncertainty but gradual improvement, leading to the relaxation of containment measures a month later. The second point, on 16 November, indicated a resurgence in infections and discussions surrounding the implementation of the “Saving Christmas plan” to mitigate the impact. The third point, on 24 November, coincided with a peak in call volumes, potentially influenced by heightened awareness campaigns and media visibility surrounding gender-based violence on the “International Day for the Elimination of Violence against Women”. The observed trend indicates a possible link between the occurrence of the pandemic and its associated containment measures with violent behaviors and the corresponding calls to the NAN helpline.

## Figures and Tables

**Table 1:** Correlation table between the study variables and number of daily calls to NAN.

Number of Daily Calls to the National Anti-Violence Number	Kendall’s Tau B	*p*
COVID-19 daily cases	0.113	0.001 **
Daily swabs	0.089	0.012 *
COVID-19 daily deaths	0.089	0.012 *
COVID-19 daily hospitalizations	0.159	<0.001 ***
COVID-19 daily intensive unit hospitalizations	0.105	0.003 **
Daily dismissed patients	0.111	**0.002**
Daily home quarantined	0.036	0.312
Daily quarantines postponed by 15 days	0.092	0.009 **
Daily quarantines postponed by 30 days	0.148	<0.001 ***

Legend. * Correlation is significant at the <0.05 level (2-tailed). ** Correlation is significant at the <0.01 level (2-tailed). *** Correlation is significant at the <0.01 level (2-tailed). The significances, according to Bonferroni corrections, are highlighted in bold.

**Table 2:** Granger causality test results (significant results).

	F-Statistic	*p*-Value	gctest Chi-Square	*p*
Daily hospitalizations Granger-caused Violence daily calls	2.05	0.087	8.19	0.084
Daily home quarantined Granger-caused Violence daily calls	2.04	0.089	8.13	0.086
Daily dismissed patient Granger-caused Violence daily calls	3.15	0.014	12.74	0.012
Violence daily calls Granger-caused SARS-CoV-2 daily cases	1.41	0.004	15.81	0.003
Violence daily calls Granger-caused SARS-CoV-2 daily deaths	0.38	0.013	12.84	0.121
Violence daily calls Granger-caused Daily swabs	1.46	≃0	27.7	≃0
Violence daily calls Granger-caused Daily hospitalizations	2.05	≃0	22.34	<0.001
Violence daily calls Granger-caused Daily intensive unit hospitalizations	1.17	0.026	11.2	0.024
Violence daily calls Granger-caused Daily dismissed patients	3.18	0.053	9.45	0.05
Violence daily calls Granger-caused Daily quarantines postponed for 15 days	1.11	0.017	12.16	0.016
Violence daily calls Granger-caused Daily quarantines postponed for 30 days	0.64	0.005	14.9	0.005

**Table 3:** Trends of 2021 daily calls to the Italian anti-violence number.

Segment	Lower Endpoint	Upper Endpoint	DPC	Lower CI	Upper CI	*t* Test	*p*-Value
1	1 (1 January)	78 (19 March)	0.5 *	0.3	0.7	4.9	<0.001
2	78 (19 March)	320 (16 November)	−0.1 *	−0.1	−0.1	−5.6	<0.001
3	320 (16 November)	328 (24 November)	18.1 *	7.7	29.5	3.5	<0.001
4	328 (24 November)	365 (31 December)	−3.6 *	−4.2	−3	−11.9	<0.001

Legend. * Indicates that the daily percent change (DPC) significantly differs from zero at the alpha = 0.05 level.

## Data Availability

Data can be requested from the corresponding author.

## Supplementary Materials

The following supporting information can be downloaded at: https://www.mdpi.com/article/10.3390/healthcare11162272/s1. Table S1: Descriptive statistics for daily calls to the National Anti-Violence Number and pandemic-related epidemiological variables in Italy during 2021.
