# Supplementary material for: Facing the Shadow Pandemic: Correlation and Trend Analyses of Violence Reports from Women to the Italian National Anti-Violence Number during 2021
Source: Healthcare (Basel). 2023 Aug 11;11(16):2272. doi: 10.3390/healthcare11162272 (PMC10454491; doi:10.3390/healthcare11162272)
Supplement: Supplementary file 1 [file healthcare-11-02272-s001.zip › healthcare-2494206-supplementary.pdf]

Table S1. Descriptive Statistics for Daily Calls to the National Anti-Violence Number and Pandemic-Related Epidemiological Variables in Italy during 2021.

Month	Mean	Std. Deviation	95% Confidence Interval for Mean		Minimum	Maximum
			Lower Bound	Upper Bound		
Daily calls to the national anti-violence number						
January	74.52	13.66	69.50	79.53	50	103
February	86.14	17.69	79.28	93	59	118
March	104.90	17.09	98.64	111.17	75	150
April	96.03	18.47	89.14	102.93	66	143
May	92.81	16.58	86.72	98.89	55	126
June	91.67	23.30	82.96	100.37	45	160
July	87.39	13.49	82.44	92.34	59	116
August	90.32	13.54	85.35	95.29	66	117
September	90.27	12.76	85.50	95.03	55	117
October	77.23	14.68	71.84	82.61	56	119
November	163	132.84	113.40	212.60	56	657
December	130.74	39.16	116.38	145.11	77	244
Total	98.73	48.08	93.78	103.68	45	657
COVID-19 daily cases						
January	14373.71	3323.74	13154.55	15592.87	8562	22211
February	13303.68	3391.86	11988.45	14618.91	7351	20499
March	20909.68	4048.39	19424.71	22394.64	12916	26824
April	14648.20	3859.57	13207.01	16089.39	7767	23649
May	6299.13	2933.12	5223.25	7375.01	1820	12965
June	1420.47	691.68	1162.19	1678.74	389	2897
July	2908.84	1959.12	2190.23	3627.45	480	6619
August	6133.94	1275.85	5665.95	6601.92	3190	7826
September	4407.33	1298.68	3922.40	4892.27	1772	6761
October	3215.61	977.56	2857.04	3574.19	1516	5335
November	8525.67	3160.12	7345.66	9705.67	2818	13764
December	35391.87	33076.28	23259.39	47524.35	9503	144243
Total	10982.93	13581.91	9584.92	12380.93	389	144243
Daily swabs						
January	195745.77	71557.10	169498.42	221993.13	67174	298010
February	266648.61	55886.25	244978.18	288319.03	142419	353704
March	315148.71	70945.81	289125.58	341171.84	156697	378463
April	289241.83	77468.81	260314.50	318169.16	102795	364804
May	244919.45	76992.74	216678.29	273160.62	86977	378202
June	180365.03	51885.06	160990.83	199739.23	75861	249988
July	192289.94	53392.74	172705.32	211874.55	73571	264860
August	209404.03	61279.58	186926.50	231881.56	74021	307643
September	280997.43	66712.38	256086.62	305908.25	120045	357491
October	380203.74	138004.05	329583.45	430824.04	114776	662000
November	502659.23	154709.86	444889.62	560428.85	146725	719972
December	671157.13	257297.45	576779.66	765534.60	217052	1224025
Total	311065.75	178377.91	292705.05	329426.45	67174	1224025
COVID-19 daily deaths						
January	463.13	96	427.92	498.34	237	649
February	327.96	73.81	299.34	356.58	192	498
March	375.71	81.70	345.74	405.68	207	551
April	382.03	107.37	341.94	422.13	217	718
May	171.65	66.12	147.39	195.90	44	305
June	47.93	23.99	38.98	56.89	12	102
July	16.03	7.04	13.45	18.61	3	31
August	37.35	17.37	30.99	43.72	2	75
September	56.67	12.01	52.18	61.15	26	73
October	38.03	11	34	42.07	14	70
November	57.60	18.69	50.62	64.58	20	90
December	115.29	34.75	102.54	128.04	43	190
Total	173.27	168.33	155.94	190.60	2	718
COVID-19 daily hospitalizations						
January	-98.55	205.01	-173.75	-23.35	-424	242
February	-52.07	186.49	-124.38	20.24	-338	351
March	340.06	224.99	257.54	422.59	-51	820

April	-341.33	325.33	-462.81	-219.85	-844	552
May	-401.87	205.51	-477.25	-326.49	-672	50
June	-162.97	91.51	-197.14	-128.80	-334	-20
July	8.32	50.65	-10.26	26.90	-75	120
August	77.45	49.22	59.40	95.51	-13	172
September	-35.13	60.42	-57.70	-12.57	-146	87
October	-14.32	53.78	-34.05	5.41	-113	106
November	82.43	47.46	64.71	100.15	11	171
December	191.06	130.84	143.07	239.06	21	503
Total	-32.88	254.91	-59.12	-6.64	-844	820
COVID-19 daily intensive unit hospitalizations						
January	-10.97	27.36	-21.01	-0.93	-64	41
February	0.57	25.95	-9.49	10.63	-69	37
March	47.71	27.96	37.45	57.97	-6	100
April	-37.57	34.94	-50.61	-24.52	-101	34
May	-50	24.44	-58.97	-41.03	-102	2
June	-26.20	16.66	-32.42	-19.98	-71	-4
July	-1.06	8.12	-4.04	1.92	-18	17
August	10.65	8.26	7.61	13.68	-5	24
September	-3.47	9.29	-6.94	0	-29	15
October	-3.16	7.33	-5.85	-0.47	-20	10
November	11.37	9	8.01	14.73	-4	31
December	18.61	13.17	13.78	23.44	-2	47
Total	-3.55	31.89	-6.83	-0.27	-102	100
Daily dismissed patients						
January	17659.26	3742.82	16286.38	19032.14	9166	27676
February	14094.68	3132.17	12880.15	15309.21	6847	19838
March	16382.13	4559.12	14709.83	18054.43	9835	32720
April	18417.70	3435	17135.05	19700.35	9323	26175
May	12659.45	3730.67	11291.03	14027.87	6358	19023
June	7462.77	9266.25	4002.69	10922.84	1336	53074
July	1702.52	619.60	1475.25	1929.79	501	3264
August	4456.94	1785.54	3802	5111.88	1250	8885
September	5809.37	1494.24	5251.41	6367.33	2892	8606
October	3557.77	753.21	3281.49	3834.05	2184	5245
November	4767.73	1651.66	4150.99	5384.47	1524	8041
December	12478.97	4888.15	10685.98	14271.96	5567	22579
Total	9929.28	6942.14	9214.71	10643.84	501	53074
Daily home quarantined						
January	-3630.10	4864.77	-5414.51	-1845.69	-14133	6901
February	-1077.11	4915.02	-2982.95	828.74	-9843	10120
March	4085.97	5451	2086.53	6085.41	-9232	11503
April	-3780.53	4323.31	-5394.89	-2166.18	-14949	4248
May	-6083.48	2340.09	-6941.84	-5225.13	-10716	398
June	-5918.60	9150.09	-9335.30	-2501.90	-51720	-538
July	1181.26	2012.51	443.06	1919.45	-2119	4389
August	1545.45	1554.67	975.19	2115.71	-3452	3946
September	-1415.30	1097.26	-1825.02	-1005.58	-3802	514
October	-365.10	1082.74	-762.25	32.05	-1979	2024
November	3633.60	1856.96	2940.20	4327	575	7345
December	22587.55	29041.06	11935.20	33239.89	3546	121203
Total	943.52	11802.85	-271.37	2158.40	-51720	121203
Daily home quarantine postponed by 15 days						
January	-2514.419	4731.04	-4179.84	-848.999	-13073.00	6901.00
February	-5209.464	3936.63	-6667.59	-3751.343	-14133.00	1398.00
March	4094.355	5178.65	2271.37	5917.344	-4755.00	11503.00
April	-1018.167	5722.45	-3065.88	1029.549	-14949.00	8834.00
May	-5468.258	3176.05	-6586.29	-4350.224	-11149.00	398.00
June	-7026.433	8838.81	-10189.30	-3863.564	-51720.00	-1974.00
July	-1809.742	2480.23	-2682.83	-936.652	-10799.00	1807.00
August	2677.516	1195.73	2256.60	3098.437	-24.00	4389.00
September	-447.600	1592.44	-1017.44	122.238	-3802.00	1907.00
October	-1192.226	830.25	-1484.49	-899.963	-2949.00	514.00
November	1470.367	1605.50	895.86	2044.878	-1809.00	4373.00
December	6599.355	2721.16	5641.45	7557.256	2563.00	13850.00
Total	-774.07	5624.64	-1353.02	-195.12	-51720.00	13850.00

Daily home quarantine postponed by 30 days

January	-5600.484	6901.54	-8029.96	-3171.005	-26557.00	5889.00
February	-3946.750	4565.05	-5637.64	-2255.864	-14133.00	6901.00
March	-1030.226	5098.65	-2825.05	764.601	-9843.00	10120.00
April	4058.333	5514.79	2084.93	6031.739	-9232.00	11503.00
May	-3897.161	4159.43	-5361.36	-2432.960	-14949.00	4248.00
June	-6382.233	2041.88	-7112.90	-5651.569	-10716.00	-2305.00
July	-5563.645	9064.55	-8754.55	-2372.742	-51720.00	-538.00
August	1438.742	1960.04	748.77	2128.716	-2119.00	4389.00
September	1370.300	1597.94	798.49	1942.106	-3452.00	3755.00
October	-1372.484	1093.89	-1757.56	-987.412	-3802.00	514.00
November	-292.167	1129.75	-696.44	112.104	-1979.00	2024.00
December	3947.742	2026.97	3234.21	4661.277	575.00	9026.00
Total	-1430.92	5657.09	-2013.21	-848.62	-51720.00	11503.00
